# Preliminary experience with airway pressure release ventilation on hemodynamics in patients with septic shock in a medical/surgical ICU

**DOI:** 10.1186/cc12053

**Published:** 2013-03-19

**Authors:** A Hussain, H Lababidi, A Mir, A AlHamoud, A Al Oheli, A Al Enezi

**Affiliations:** 1 King Fahd Medical City, Riyadh, Saudi Arabia

## Introduction

Airway pressure release ventilation (APRV) allows spontaneous breathing throughout the ventilation cycle. It increases venous return and cardiac index, which will significantly improve organ perfusion. This is important in septic shock patients to prevent extrathoracic organ system failure secondary to poor perfusion. Benefits of APRV with cardiovascular changes are noticed in patients with acute lung injury and acute respiratory distress syndrome. It is not well established whether applying APRV will improve the survival outcome for septic shock patients. The primary outcome is whether the use of APRV in septic shock patients restores hemodynamic stability earlier than the CMV mode. The secondary hypothesis is whether the use of APRV in septic shock patients improves their survival in the ICU.

## Methods

After Institutional Review Board approval, we retrospectively analyzed the clinical data of 129 septic shock patients who received ventilator support between January and December 2011 at a tertiary care hospital. The Cox proportional hazards model was used in adjusting potential confounding factors. The nonparametric Wilcoxon rank sum test was used to assess significant outcome differences between groups. Time to event/survival data will be analyzed using Kaplan-Meier methods. These analyses were accomplished using SAS, version 9.3.

## Results

Among the 187 patients, 58 were excluded as per the exclusion criteria: incomplete data (*n *= 28), do not resuscitate (*n *= 16), ICU readmission (*n *= 12) and head injury (*n *= 4). Finally, 129 patients were included, from these 91 received CMV and 38 received APRV. At the beginning of the study, there were no differences between the groups in relation to hemodynamic parameters. Reversal of shock achieved in less than 72 hours was statistically significant between the groups (APRV, *n *= 16 (42%) and CMV, *n *= 8 (9%), *P *= 0.0101). The proportion of patients recovering from septic shock after initiation of ventilator therapy was higher in APRV than the CMV group (72% vs. 49%, respectively, *P <*0.0001). The mortality rate was significantly higher in CMV (*n *= 46, 51%) as compared with APRV (*n *= 11, 29%) (*P *= 0.022).

## Conclusion

The use of APRV in septic shock patients restores hemodynamic stability earlier than the CMV mode. There was a significant improvement in ICU survival using APRV over CMV. Early initiation of APRV in ventilated septic shock patients was associated with a decrease in ICU mortality.

